# Analysis of Drug Resistance Determinants in *Klebsiella pneumoniae* Isolates from a Tertiary-Care Hospital in Beijing, China

**DOI:** 10.1371/journal.pone.0042280

**Published:** 2012-07-31

**Authors:** Bin Li, Yong Yi, Qi Wang, Patrick C. Y. Woo, Lin Tan, Hua Jing, George F. Gao, Cui Hua Liu

**Affiliations:** 1 Chinese Academy of Sciences Key Laboratory of Pathogenic Microbiology and Immunology, Institute of Microbiology, Chinese Academy of Sciences, Beijing, China; 2 The State Key Laboratory of Microbial Technology, Shandong University, Jinan, China; 3 The 306 Hospital, Beijing, China; 4 State Key Laboratory of Emerging Infectious Diseases, Department of Microbiology, The University of Hong Kong, Hong Kong Special Administrative Region, China; St. Petersburg Pasteur Institute, Russian Federation

## Abstract

**Background:**

The rates of multidrug-resistant (MDR), extensively drug-resistant (XDR) and pandrug-resistant (PDR) isolates among *Enterobacteriaceae* isolates, particularly *Klebsiella pneumoniae*, have risen substantially worldwide.

**Methodology/Principal Findings:**

To better understand the molecular mechanisms of drug resistance in K. pneumoniae, we analyzed the drug resistance determinants for K. pneumoniae isolates collected from the 306 Hospital, a tertiary-care hospital in Beijing, China, for the period of September 1, 2010-October 31, 2011. Drug susceptibility testing, PCR amplification and sequencing of the drug resistance determinants were performed. Conjugation experiments were conducted to examine the natural ability of drug resistance to disseminate among Enterobacteriaceae strains using a sodium azide-resistant Escherichia coli J53 strain as a recipient. Among the 223 consecutive non-repetitive K. pneumoniae isolates included in this study, 101 (45.3%) were extended-spectrum beta-lactamases (ESBLs) positive. The rates of MDR, XDR, and PDR isolates were 61.4% (n = 137), 22.0% (n = 49), and 1.8% (n = 4), respectively. Among the tested drug resistance-associated genes, the following ones were detected at relatively high rates *bla*_CTX-M-10_ (80, 35.9%), *aacC2* (73, 32.7%), *dhfr* (62, 27.8%), *qnrS* (58, 26.0%), *aacA4* (57, 25.6%), *aadA1* (56, 25.1%). Results from conjugation experiments indicate that many of the drug resistance genes were transmissible.

**Conclusions/Significance:**

Our data give a “snapshot” of the complex genetic background responsible for drug resistance in *K. pneumoniae* in China and demonstrate that a high degree of awareness and monitoring of those drug resistance determinants are urgently needed in order to better control the emergence and transmission of drug-resistant *K. pneumoniae* isolates in hospital settings.

## Introduction

The emergence and rapid spread of drug-resistant *Klebsiella pneumoniae* isolates is becoming a serious antibiotic management problem and causing a great concern worldwide [1]–[6]. For example, by late 2009, the number of unique protein sequences for beta-lactamases exceeded 890 (http://www.lahey.org/Studies) [7]. There is an increasing recognition of isolates producing newer beta-lactamases including the extended-spectrum beta-lactamase (ESBL), carbapenem-hydrolyzing enzymes (e.g., *K. penumoniae* carbapenemase [KPC] types and the metallo-beta-lactamases [MBLs]) [8]–[18]. Since the production of newer beta-lactamases is frequently accompanied by broad-spectrum resistance, the ESBL positivity together with the existence of newer beta-lactamases should be monitored closely as the emergence of those highly drug-resistant *K. pneumoniae* strains will pose a serious impact on the remaining therapeutic options [19]–[22]. In a study based on the Tigecycline Evaluation and Surveillance Trial (TEST) global surveillance database, the rate of ESBL production was highest among the *K. pneumoniae* isolates collected in Latin America, followed by Asia/Pacific Rim, Europe, and North America (44.0%, 22.4%, 13.3% and 7.5%, respectively) [23]. Thus the potential of drug resistant *K. pneumoniae* to be a global health problem is great and more intensive surveillance and more in-depth investigation into the molecular mechanisms of drug resistance in *K. pneumoniae* isolates are necessary in order to provide information for the development of effective molecular diagnostic methods and novel drugs against *K. pneumonia*e infection.

In the face of increasing resistance among multidrug-resistant (MDR) gram-negative organisms for which no adequate therapeutic options exist, a joint initiative by the European Centre for Disease Prevention and Control (ECDC) and the Centers for Disease Control and Prevention (CDC) recently created a standardized international definitions for MDR, extensively drug-resistant (XDR) and pandrug-resistant (PDR) with an aim to enhance the comparability of data and promote better comprehension of the problem of highly drug-resistant bacteria [24]. MDR was defined as acquired non-susceptibility to at least one agent in three or more antimicrobial categories, XDR was defined as non-susceptibility to at least one agent in all but two or fewer antimicrobial categories (i.e. bacterial isolates remain susceptible to only one or two categories) and PDR was defined as non-susceptibility to all agents in all antimicrobial categories [24]. Though there are already many reports of drug-resistant *K. pneumoniae* worldwide, the extent of MDR, XDR and PDR *K. pneumoniae* isolates among patients is largely unknown. We thus in this study sought to determine the prevalence of MDR, XDR and PDR strains and to analyze the drug resistance determinants for *K. pneumoniae* isolates collected from patients being treated in the 306 Hospital, a tertiary-care hospital in Beijing, China, for the period of September 1, 2010-October 31, 2011 with an aim to better understand the current situation as well as the genetic background of the drug resistant *K. pneumoniae* isolates from hospital settings.

## Methods

### Ethics Statement

All of the investigation protocols in this study were approved by the institutional ethics committee of the 306 Hospital, Beijing, China. Written consent was given by the patients for their information to be stored in the hospital database and used for research. Permission for using the information in the medical records of the patients for research purposes was obtained from the 306 Hospital. The Institute ethics committee of the 306 Hospital reviewed that relevant ethical issues in this study were well considered.

### Study Population, Bacterial Isolate Identification, and Drug Susceptibility Testing

This is a prospective surveillance study. Consecutive *K. pneumoniae* isolates were collected from unique patients being treated in the 306 Hospital in Beijing, China (which is a 1,100-bed tertiary-care hospital serving approximately 25,000 in-patients per year) for the period of September 1, 2010-October 31, 2011. In the case of duplicate patient samples, the first collected isolate was chosen. All strains were cultured in Luria–Bertani (LB) medium. The *K. pneumoniae* strains were confirmed by phenotypic tests and 16 S rDNA sequencing. Drug susceptibility testing (DST) for the *K. pneumoniae* strains was performed using the bioMérieux VITEK-2 AST-GN13 system following manufacturer’s instructions. The following 18 drugs were tested: ampicillin (AMP), piperacillin/tazobactam (TZP), ampicillin/sulbactam (SAM), cefazolin (CFZ), ceftriaxone (CRO), ceftazidime (CAZ), cefepime (FEP), cefotetan (CTT), ertapenem (ETP), imipenem (IMP), aztreonam (ATM), ciprofloxacin (CIP), levofloxacin (LVX), gentamicin (GM), tobramycin (TOB), amikacin (AMK), trimethoprim-sulfamethoxazole (SXT), furadantin (FD). The ESBLs were detected by the bioMérieux VITEK-2 AST-GN13 test (which is claimed to be a confirmatory ESBL test). In some cases, the ESBL positivity was further confirmed by the double disk diffusion method [25]. *Escherichia coli* strains ATCC 25922 and ATCC 35218, *K. pneumoniae* strain ATCC 700603 and *Pseudomonas aeruginosa* strain ATCC 27853 were used as quality control strains for the DST. Clinical records of patients from whom the *K. pneumoniae* isolates were obtained were reviewed retrospectively.

### PCR Amplification and Sequencing

Genomic DNA was extracted using DNeasy Tissue kit (Qiagen; Valencia, CA, USA). Drug resistance-associated genes were detected by PCR and sequencing using 37 pairs of primers listed in Table 1. Direct sequencing of positive amplicons was conducted. The primers were synthesized by the Beijing Genomics Institute (BGI, China). PCR was performed in a 50-µL reaction mixture consisting of 5 µL of 10×PCR buffer, 2.5 units of Taq DNA polymerase (Takara), 0.2 mM of dNTPs, 0.4 µM each of the primer, and 1 µL chromosomal DNA. All reaction mixtures were subjected to 30 cycles of 94°C for 1 min, 55°C for 1 min, and 72°C for 2 min. PCR products were purified and sequenced bi-directionally with the same primers used for PCR by the Beijing Genomics Institute (BGI, China). DNA sequences were annotated using the BLAST program at http://www.ncbi.nlm.nih.gov. Mutations in the *gyrA* and *parC* genes were identified by comparing the DNA sequences with *gyrA* and *parC* sequences of the *K. pneumoniae* (GenBank accession numbers DQ673325 and NC009648 for *gyrA* and *parC*, respectively).

**Table 1:** Primers used for PCR and sequencing of drug resistance-associated genes from *K. pneumoniae* isolates.

Target genes	Primer sequence (5′ to 3′)		Ampliconsize (bp)	Source ofreference
	Forward	Reverse		
*bla*_CTX-M-1_	GGT TAA AAA ATC ACT GCG TC	TTA CAA ACC GTC GGT GAC GA	876	[2]
*bla*_CTX-M-2_	ATG ATG ACT CAG AGC ATT CG	TTA TTG CAT CAG AAA CCG TG	876	[29]
*bla*_CTX-M-3_	GTT GTT GTT ATT TCG TAT CTT CC	CGA TAA ACA AAA ACG GAA TG	934	[10]
*bla*_CTX-M-8_	ATG ATG AGA CAT CGC GTT AAG	CGG TGA CGA TTT TCG CGG CAG	864	[61]
*bla*_CTX-M-9_	GTG ACA AAG AGA GTG CAA CGG	ATG ATT CTC GCC GCT GAA GCC	850	[31]
*bla*_CTX-M-10_	GCA GCA CCA GTA AAG TGA TGG	GCG ATA TCG TTG GTG GTA CC	873	[30]
*bla*_CTX-M-14_	ACA ATG ACG CTG GCA GAA CTG	TTA CAG CCC TTC GGC GAT GA	512	[64]
*bla*_CTX-M-25_	CAC ACG AAT TGA ATG TTC AG	TCA CTC CAC ATG GTG AGT	924	[61]
*bla*_SHV-group_	TTT ATC GGC CYT CAC TCA AGG	GCT GCG GGC CGG ATA ACG	896	[32]
*bla*_TEM_	KAC AAT AAC CCT GRT AAA TGC	AGT ATA TAT GAG TAA ACT TGG	899	[32]
*bla*_KPC_	ATG TCA CTG TAT CGC CGT CT	TTT TCA GAG CCT TAC TGC CC	882	[8]
*bla*_NDM_	GGT TTG GCG ATC TGG TTT TC	CGG AAT GGC TCA TCA CGA TC	621	[51]
*bla*_IMP_	GGA ATA GAG TGG CTT AAT TCT C	CCA AAC CAC TAC GTT ATC	624	[27]
*bla*_VIM_	GGT CTC ATT GTC CGT GAT GGT GAT GAG	CTC GAT GAG AGT CCT TCT AGA G	271	[27]
*bla*_OXA-48_	TTG GTG GCA TCG ATT ATC GG	GAG CAC TTC TTT TGT GAT GGC	743	[47]
*bla*_CMY_	TGG CCA GAA CTG ACA GGC AAA	TTT CTC CTG AAC GTG GCT GGC	462	[65]
*bla*_DHA_	AAC TTT CAC AGG TGT GCT GGG T	CCG TAC GCA TAC TGG CTT TGC	405	[65]
*bla*_FOX_	AAC ATG GGG TAT CAG GGA GAT G	CAA AGC GCG TAA CCG GAT TGG	190	[65]
*dhfr*	GCC AAT CGG GTT ATT GGC AA	TGG GAA GAA GGC GTC ACC CTC	357	[15]
*qnrA*	ATT TCT CAC GCC AGG ATT TG	GAT CGG CAA AGG TTA GGT CA	627	[62]
*qnrB*	GAT CGT GAA AGC CAG AAA GG	ACG ATG CCT GGT AGT TGT CC	469	[62]
*qnrC*	GGG TTG TAC ATT TAT TGA ATC G	CAC CTA CCC ATT TAT TTT CA	307	[34]
*qnrD*	CGA GAT CAA TTTA CGG GGA ATA	AAC AAG CTG AAG CGC CTG	533	[33]
*qnrS*	ACG ACA TTC GTC AAC TGC AA	TAA ATT GGC ACC CTG TAG GC	417	[28]
*aac(6′)-Ib-cr*	TTG CGA TGC TCT ATG AGT GGC TA	CTC GAA TGC CTG GCG TGT TT	482	[35]
*qepA*	AAC TGC TTG AGC CCG TAG AT	GTC TAC GCC ATG GAC CTC AC	596	[34]
*gyrA*	CGA CCT TGC GAG AGA AAT	GTT CCA TCA GCC CTT CAA	626	[54]
*parC*	TAC GTC ATC ATG GAC AGG	GCC ACT TCA CGC AGG TTG	460	[37]
*aacA4*	ATG ACT GAG CAT GAC CTT GCG	TTA GGC ATC ACT GCG TGT TCG	540	[38]
*aacC1*	ATG GGC ATC ATT CGC ACA TGT AGG	TTA GGT GGC GGT ACT TGG GTC	873	[38]
*aacC2*	ATG CAT ACG CGG AAG GCA ATA AC	CTA ACC GGA AGG CTC GCA AG	861	[38]
*aadA1*	ATG AGG GAA GCG GTG ATC G	TTA TTT GCC GAC TAC CTT GGT G	792	[38]
*aadB*	ATG GAC ACA ACG CAG GTC GC	TTA GGC CGC ATA TCG CGA CC	534	[38]
*aphA6*	ATG GAA TTG CCC AAT ATT ATT C	TCA ATT CAA TTC ATC AAG TTT TA	781	[38]
*armA*	AGG TTG TTT CCA TTT CTG AG	TCT CTT CAT TCC CTT CTC C	591	[57]
*rmtB*	CCC AAA CAG ACC GTA GAG GC	CTC AAA CTC GGC GGG CAA GC	585	[57]
*Integron I*	GGC ATC CAA GCA CAA G	AAG CAG ACT TGA CCT GA	Variable	[63]

### Conjugation Experiments, Plasmid Analysis, and MLST Analysis

Transfer of resistance genes by conjugation experiments were carried out in LB broth using clinical isolates as donors and the *E. coli* J53AzR as the recipient as described previously [26]. Cultures of donor and recipient cells in logarithmic phase (0.5 mL each) were added to 4 mL of fresh LB broth and incubated overnight without shaking. Transconjugants were selected on LB plates containing 100 µg/mL sodium azide for counterselection and 100 µg/mL ampicillin to select for plasmid-encoded resistance. The drugs tested were purchased from Sigma Chemical Co. Plasmid DNA from the *K. pneumonia*e donor strains and *E. coli* transconjugants were prepared using the Plasmid Maxprep Kit (Vigorous Biotechnology, Beijing, China) and were separated on 0.7% agarose gels. Genotyping was determined by MLST analysis. MLST with seven genes (*gapA*, *infB*, *mdh*, *pgi*, *phoE*, *rpoB* and *tonB*) was performed on isolates according to the protocol described on the *K. pneumoniae* MLST website (www.pasteur.fr/mlst). Sequence types (STs) were assigned by using the MLST database (www.pasteur.fr/mlst/Kpneumoniae.html).

### Statistical Analysis

SPSS software (version 15.0) was used for data analysis. Categorical variables were compared with the chi-square test or Fisher’s exact test. A *p* value of <0.05 was considered to be statistically significant.

## Results

### Demographic and Clinical Characteristics of the Patients

From September 1, 2010 to October 31, 2011, a total of 223 non-repetitive patients at the 306 Hospital who had *K. pneumoniae* isolates were subjected to DST using 18 antibiotics. Among which, 137 (61.4%) were MDR isolates, 49 (22.0%) were XDR isolates, 4 (1.8%) were PDR isolates, and 33 (14.8%) were other types of isolates. The proportion of the male and female were 73.5% (n = 164) and 26.5% (n = 59), respectively. Sixty-eight (30.5%) of the patients were Beijing residents and the rest were from other provinces of China (non-Beijing residents). The median (±SD) age of the patients was 74.0±20.3 years (range 1.0–98.0 years). The majority of the patients were from medical ward (97, 43.5%) and intensive care unit (75, 33.6%). The main source of the specimens was sputum (168, 75.3%). The proportion of the ESBL positive cases was 45.3% (n = 101). The proportion of XDR (42, 41.6%) and PDR (4, 4.0%) cases was significantly higher among patients whose isolates were ESBL positive as compared with those whose isolates were ESBL negative. In addition, the proportion of MDR cases (83, 68.0%) and other types of cases (32, 26.2%) was significantly higher in patients with ESBL-negative isolates than that observed for XDR (7, 5.7%) and PDR cases (0). The detailed information on relevant demographic and clinical characteristics of the study population is summarized in Table 2.

**Table 2:** Demographic and clinical characteristics of the patients.

Characteristics	Total n = 223 (%)	Patients infected with MDR isolates n = 137 (%)	Patients infected with XDR isolates n = 49 (%)	Patients infected with PDR isolates n = 4 (%)	Patients infected with other types of isolates n = 33 (%)	*P* value
Gender						0.991
Male	164 (73.5)	101 (73.7)	35 (71.4)	4 (100)	24 (72.7)	
Female	59 (26.5)	36 (26.3)	14 (28.6)	0	9 (27.3)	
Age group, years						0.400
<18	7 (3.1)	6 (4.4)	0	0	1 (3.0)	
18–64	64 (28.7)	39 (28.5)	11 (22.4)	2 (50.0)	12 (36.4)	
>64	152 (68.2)	92 (67.2)	38 (77.6)	2 (50.0)	20 (60.6)	
Residence situation						0.862
Beijing resident	68 (30.5)	40 (29.2)	17 (34.7)	0	11 (33.3)	
Non-Beijing resident	155 (69.5)	97 (70.8)	32 (65.3)	4 (100.0)	22 (66.7)	
Hospital location						0.199
Emergency room	9 (4.0)	7 (5.1)	2 (4.1)	0	0	
Intensive care unit	75 (33.6)	51 (37.2)	17 (34.7)	0	7 (21.2)	
Medical ward	97 (43.5)	59 (43.1)	18 (36.7)	2 (50.0)	18 (54.5)	
Surgical ward	42 (18.8)	20 (14.6)	12 (24.5)	2 (50.0)	8 (24.2)	
Sources of specimens						0.187
Sputum	168 (75.3)	108 (78.8)	34 (69.4)	3 (75.0)	23 (69.7)	
Urine	14 (6.3)	7 (5.1)	7 (14.3)	0	0	
Throat or nose swabs	21 (9.4)	11 (8.0)	2 (4.1)	0	8 (24.2)	
Catheters	3 (1.3)	2 (1.5)	1 (2.0)	0	0	
Blood	10 (4.5)	4 (2.9)	3 (6.1)	1(25.0)	2 (6.1)	
Puncture fluid	1 (0.4)	1 (0.7)	0	0	0	
Drainage fluid	1 (0.4)	1 (0.7)	0	0	0	
Pleural effusion	2 (0.9)	1 (0.7)	1 (2.0)	0	0	
Plus	1 (0.4)	1 (0.7)	0	0	0	
Bile	2 (0.9)	1 (0.7)	1 (2.0)	0	0	
ESBL						<0.001
Positive	101 (45.3)	54 (39.4)	42 (85.7)	4(100.0)	1 (3.0)	
Negative	122 (54.7)	83 (60.6)	7 (14.3)	0	32 (97.0)	

### Drug Susceptibility Patterns of the *K. pneumoniae* Isolates

DST was conducted for 223 *K. pneumoniae* isolates and the detailed information on resistance rates to all tested drugs are listed in Table 3. The highest resistance rate was observed for AMP, reaching 99.6% (n = 222), followed by resistance to FD (190, 85.2%), CTT (153, 68.6%), SXT (118, 52.9%), SAM (115, 51.6%), CFZ (114, 51.1%), CRO (110, 49.3%), CAZ (110, 49.3%), ATM (109, 48.9%), and TOB (109, 48.9%). The two carbapenems tested including ETP and IMP exhibited relatively lower resistance rates (7.2% and 5.8%, respectively). Notably, the rates of resistance to most drugs were much higher among ESBL positive isolates than ESBL negative isolates.

**Table 3:** Drug resistance rates of *K. pneumoniae* isolates.

Antimicrobial category	Drugs^a^	Range (µg/mL)	MIC_50_ (µg/mL)	MIC_90_ (µg/mL)	ESBL positive n = 101 (%)	ESBL negative n = 122 (%)	Total n = 223 (%)
Penicillins (99.6%, 222/223)	AMP	≤2–≧32	≥32	≥32	101 (100)	121 (99.2)	222 (99.6)
Antipseudomonal penicillins + beta-lactamase inhibitors (22.4%, 50/223)	TZP	≤4–≧128	≤4	≥128	29 (28.7)	21 (17.2)	50 (22.4)
Penicillins + beta-lactamase inhibitors (51.6%, 115/223)	SAM	≤2–≧32	16	≥32	88 (87.1)	27 (22.1)	115 (51.6)
1st and 2nd generation cephalosporins (51.1%, 114/223)	CFZ	≤4–≧64	32	≥64	96 (95.0)	18 (14.8)	114 (51.1)
3rd and 4th generation cephalosporins (49.3%, 110/223)	CRO	≤1–≧64	≤1	≥64	95 (94.1)	15 (12.3)	110 (49.3)
	CAZ	≤1–≧64	≤1	≥64	95 (94.1)	15 (12.3)	110 (49.3)
	FEP	≤1–≧64	≤1	≥64	96 (95.0)	12 (9.8)	108 (48.4)
Cephamycins (68.6%, 153/223)	CTT	≤4–≧64	≤4	≤4	43 (42.6)	110 (90.2)	153 (68.6)
Carbapenems (7.2%, 16/223)	ETP	≤0.5–≧8	≤0.5	≤0.5	5 (5.0)	11 (9.0)	16 (7.2)
	IMP	≤1–≧16	≤1	≤1	3 (3.0)	10 (8.2)	13 (5.8)
Monobactams (48.9%, 109/223)	ATM	≤1–≧64	≤1	≥64	96 (95.0)	13 (10.7)	109 (48.9)
Fluoroquinolones (40.4%, 90/223)	CIP	≤0.25–≧4	1	≥4	63 (62.4)	27 (22.1)	90 (40.4)
	LVX	≤0.25–≧8	1	≥8	63 (62.4)	27 (22.1)	90 (40.4)
Aminoglycosides (49.3%, 110/223)	GM	≤1–≧16	≤1	≥16	70 (69.3)	22 (18.0)	92 (41.3)
	TOB	≤1–≧16	≤1	≥16	82 (81.2)	27 (22.1)	109 (48.9)
	AMK	≤2–≧64	≤2	16	39 (38.6)	15 (12.3)	54 (24.2)
Folate pathway inhibitors (52.9%, 118/223)	SXT	≤20–≧320	≥320	≥320	90 (89.1)	28 (23.0)	118 (52.9)
Nitrofurantoin (85.2%, 190/223)	FD	≤16–≧512	64	≥512	89 (88.1)	101 (82.8)	190 (85.2)

a Abbreviation of drugs: AMP, Ampicillin; TZP, Piperacillin/Tazobactam; SAM, Ampicillin/Sulbactam; CFZ, Cefazolin; CRO, Ceftriaxone; CAZ, Ceftazidime; FEP, Cefepime; CTT, Cefotetan; ETP, Ertapenem; IMP, Imipenem; ATM, Aztreonam; CIP, Ciprofloxacin; LVX, Levofloxacin; GM, Gentamycin; TOB, Tobramycin; AMK, Amikacin; SXT, Trimethoprim-Sulfamethoxazole; FD, Furadantin.

### Drug Resistance Determinants of the *K. pneumonia*e Isolates

PCR and sequencing analysis were conducted for 223 *K. pneumoniae* isolates to analyze ESBL genes as well as drug resistance determinants conferring resistance to carbapenems, folate pathway inhibitors, fluoroquinolones, and aminoglycosides. The detailed information on the percentage of drug resistance-associated genes detected in *K. pneumoniae* isolates were summarized in Table 4 and Table 5. The percentage of isolates with 8 or more drug resistance-associated genes was 24.2% (n = 54). Among the beta-lactamase genes, the most frequently detected ones include: *bla*_CTX-M-10_ (80, 35.9%), *bla*_SHV-1_ (55, 24.7%), *bla*_SHV-11_ (47, 21.1%), *bla*_CTX-M-1_ (37, 16.6%), *bla*_CTX-M-14_ (37, 16.6%), *bla*_CTX-M-15_ (34, 15.2%) and *bla*_CTX-M-3_ (32, 14.3%). Except for *bla*_NDM_, all the other 4 examined carbapenemase genes including *bla*_KPC-2_ (3, 1.3%), *bla*_IMP_ (1, 0.4%), *bla*_VIM_ (1, 0.4%), and *bla*_OXA-48_ (5, 2.2%) were detected in this study. The prevalence of AmpC beta-lactamases including *bla*_CMY-2_, *bla*_DHA-1_ and *bla*_FOX_ were 3.1%, 4.0% and 0, respectively in this study. Among the 7 plasmid-encoded fluoroquinolone resistance-associated genes including *qnrA, qnrB, qnrC, qnrD, qnrS, aac(6′)-Ib-cr,* and *qepA*, the highest rates were observed for *qnrS* (58, 26.0%) and *aac(6′)-Ib-cr* (53, 23.8%). In addition, *gyrA* gene mutations including T247A (Ser83Ile) (21, 9.4%), C248T (Ser83Phe) (15, 6.7%), and A260C (Asp87Ala) (16, 7.2%) were identified. No mutations were detected in *parC* gene. Among the aminoglycosides resistance-associated genes, the highest rates were observed for *aacC2* (73, 32.7%), *aacA4* (57, 25.6%), and *aadA1* (56, 25.1%). The prevalence of the plasmid-encoded 16 S rRNA methylases *armA* and *rmtB* were detected to be 5.8% and 3.6%, respectively. Class 1 integrons were detected in 47.5% (n = 106) of the isolates. In order to evaluate the correlation between phenotypic and genotypic drug resistance profiles, we also calculated the proportion of antibiotic resistance-associated genes among the phenotypic resistant isolates as well as the phenotypic susceptible isolates. We found that while some of the previously reported resistance-associated genes were indeed detected at relatively higher rates among corresponding phenotypic resistant isolates, some others were detected in very low proportion of the phenotypic resistant isolates. In addition, some of the resistance-associated genes were also detected in a sizable proportion of the phenotypic susceptible isolates.

**Table 4:** Percentage of beta-lactamase antibiotics resistance-associated genes detected in *K. pneumoniae* isolates.

Target antimicrobial category	Resistance-associated genes	Resistance-associated genes detected in phenotypic resistant isolates, n/N^a^ (%)	Resistance-associated genes detected in phenotypic susceptible isolates, n/N^b^ (%)	Resistance-associated genes detected in all isolates, n/N^c^ (%)
Antipseudomonal penicillins + beta-lactamase inhibitors, penicillins + beta-lactamase inhibitors, 1st and 2nd generation cephalosporins, 3rd and 4th generation cephalosporins, cephamycins (n = 204)	*bla*_CTX-M-1_	35/204 (17.2)	2/19 (10.5)	37/223 (16.6)
	*bla*_CTX-M-2_	1/204 (0.5)	1/19 (5.3)	2/223 (0.9)
	*bla*_CTX-M-3_	31/204 (15.2)	1/19 (5.3)	32/223 (14.3)
	*bla*_CTX-M-8_	20/204 (9.8)	2/19 (10.5)	22/223 (9.9)
	*bla*_CTX-M-9_	18/204 (8.8)	2/19 (10.5)	20/223 (9.0)
	*bla*_CTX-M-14_	37/204 (18.1)	0	37/223 (16.6)
	*bla*_CTX-M-15_	34/204 (16.7)	0	34/223 (15.2)
	*bla*_CTX-M-10_	76/204 (37.3)	4/19 (21.1)	80/223 (35.9)
	*bla*_CTX-M-25_	2/204 (1.0)	1/19 (5.3)	3/223 (1.3)
	*bla*_CTX-M-55_	3/204 (1.5)	0	3/223 (1.3)
	*bla*_SHV-1_	55/204 (27.0)	0	55/223 (24.7)
	*bla*_SHV-2_	2/204 (1.0)	0	2/223 (0.9)
	*bla*_SHV-11_	44/204 (21.6)	3/19 (15.8)	47/223 (21.1)
	*bla*_SHV-85_	15/204 (7.4)	0	15/223 (6.7)
	*bla*_TEM-1_	19/204 (9.3)	0	19/223 (8.5)
	*bla*_TEM-186_	3/204 (1.5)	0	3/223 (1.3)
	*bla*_CMY-2_	7/204 (3.4)	0	7/223 (3.1)
	*bla*_DHA-1_	9/204 (4.4)	0	9/223 (4.0)
	*bla*_FOX_	0	0	0
Carbapenems (n = 16)	*bla*_KPC-2_	3/16 (18.8)	0	3/223 (1.3)
	*bla*_NDM-1_	0	0	0
	*bla*_IMP_	1/16 (6.3)	0	1/223 (0.4)
	*bla*_VIM_	1/16 (6.3)	0	1/223 (0.4)
	*bla*_OXA-48_	5/16 (31.3)	0	5/223 (2.2)

a **n/N**: No. of designated drug resistance-associated genes/No. of isolates resistant to the corresponding drugs.

b **n/N**: No. of designated drug resistance-associated genes/No. of isolates susceptible to the corresponding drugs.

c **n/N**: No. of designated drug resistance-associated genes/No. of all isolates.

**Table 5:** Percentage of non-beta-lactamase antibiotics resistance-associated genes detected in *K. pneumoniae* isolates.

Target antimicrobial category	Resistance-associated genes	Resistance-associated genes detected in phenotypic resistant isolates, n/N^a^ (%)	Resistance-associated genes detected in phenotypic susceptible isolates, n/N^b^ (%)	Resistance-associated genes detected in all isolates, n/N^c^ (%)
Folate pathway inhibitors (n = 118)	*Dhfr*	52/118(44.1)	10/105 (9.5)	62/223 (27.8)
Fluoroquinolones (n = 90)	*qnrA*	1/90 (1.1)	1/133 (0.8)	2/223 (0.9)
	*qnrB*	33/90 (36.7)	6/133 (4.5)	39/223 (17.5)
	*qnrC*	1/90 (1.1)	1/133 (0.8)	2/223 (0.9)
	*qnrD*	23/90 (25.6)	12/133 (9.0)	35/223 (15.7)
	*qnrS*	22/90 (24.4)	36/133 (27.1)	58/223 (26.0)
	*aac(6′)-Ib-cr*	45/90 (50.0)	8/133 (6.0)	53/223 (23.8)
	*qepA*	1/90 (1.1)	1/133 (0.8)	2/223 (0.9)
	*gyrA* mutations			
	T247A(Ser83Ile)	18/90 (20.0)	3/133 (2.3)	21/223 (9.4)
	C248T (Ser83Phe)	12/90 (13.3)	3/133 (2.3)	15/223 (6.7)
	A260C(Asp87Ala)	13/90 (14.4)	3/133 (2.3)	16/223 (7.2)
	*parC* mutations	None	None	None
Aminoglycosides (n = 110)	*aacA4*	50/110 (45.5)	7/113 (6.2)	57/223 (25.6)
	*aacC1*	3/110 (2.7)	0	3/223 (1.3)
	*aacC2*	66/110 (60.0)	7/113 (6.2)	73/223 (32.7)
	*aadA1*	49/110 (44.5)	7/113 (6.2)	56/223 (25.1)
	*aadB*	4/110 (3.6)	0	4/223 (1.8)
	*aphA6*	1/110 (0.9)	0	1/223 (0.4)
	*armA*	13/110 (11.8)	0	13/223 (5.8)
	*rmtB*	8/110 (7.3)	0	8/223 (3.6)

a **n/N**: No. of designated drug resistance-associated genes/No. of isolates resistant to the corresponding drugs.

b **n/N**: No. of designated drug resistance-associated genes/No. of isolates susceptible to the corresponding drugs.

c **n/N**: No. of designated drug resistance-associated genes/No. of all isolates.

### Characteristics of Carbapenem-resistant *K. pneumoniae* Isolates

Among the 223 isolates, 16 were detected to be carbapenem-resistant and were used for further characterization. Most of the patients from whom the isolates obtained were male 87.5% (14/16) and aged patients (all were 56 years old or above). Among the 14 patients whose treatment outcome information was available, 6 died. Although carbapenemase genes were detected only in 7 of the 16 isolates, the majority of them exhibited resistance to a high number of drugs and contained a variety of corresponding drug resistance-associated genes. The proportion of MDR, XDR and PDR were 12.5% (n = 2), 62.5% (n = 10), 25.0% (n = 4), respectively. The more detailed characteristics of those carbapenem-resistant isolates are shown in Table S1.

### Transmissibility of Drug Resistance of ESBL Positive MDR, XDR and PDR *K. pneumoniae* Isolates

**Figure 1:**
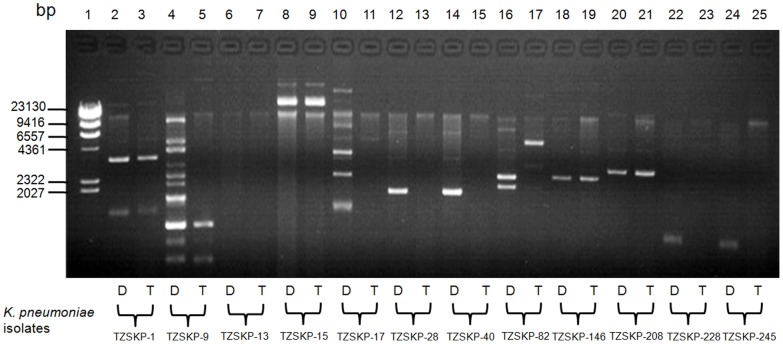
Analysis of plasmid DNA from *K. pneumoniae* parental donor strains (Designated D) and derived *E. coli* transconjugants (Designated T) by agarose gel electrophoresis. Lanes: 1, molecular marker (λ-Hind β digest DNA Marker, TaKaRa Code: D3403A); 2, TZSKP-1; 3, Transconjugant from TZSKP-1; 4, TZSKP-9; 5, Transconjugant from TZSKP-9; 6, TZSKP-13; 7, Transconjugant from TZSKP-13; 8, TZSKP-15; 9, Transconjugant from TZSKP-15; 10, TZSKP-17; 11, Transconjugant from TZSKP-17; 12, TZSKP-28; 13, Transconjugant from TZSKP-28; 14, TZSKP-40; 15, Transconjugant from TZSKP-40; 16, TZSKP-82; 17, Transconjugant from TZSKP-82; 18, TZSKP-146; 19, Transconjugant from TZSKP-146; 20, TZSKP-208; 21, Transconjugant from TZSKP-208; 22, TZSKP-228; 23, Transconjugant from TZSKP-228; 24, TZSKP-245; 25, Transconjugant from TZSKP-245.

**Figure 2:**
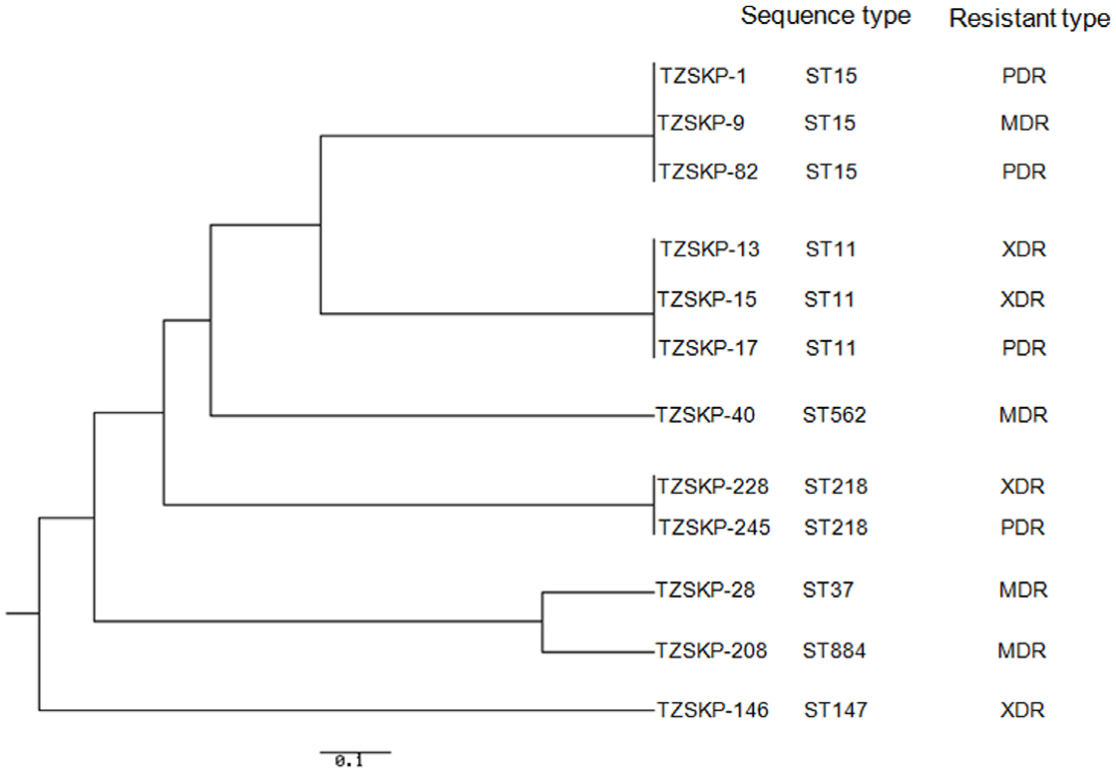
Phylogenetic tree for the 7 housekeeping loci in *K. pneumoniae* constructed using the UPGMA method, displaying the clonal relationship among the *K. pneumoniae* isolates.

Twelve *K. pneumoniae* isolates including 4 MDR, 4 XDR, and 4 PDR isolates were selected to test the natural transmissibility of antibiotic resistance by conjugation experiments. As shown in Table 6, the resistance to various drugs and the corresponding resistance-associated genes were transferred in all tested isolates, though to different extent. The most frequently transferred genes include *aac(6′)-Ib-cr* (6/7, 85.7%), *bla*_CTX-M-14_ (4/5, 80.0%), *bla*_CTX-M-9_ (2/3, 66.7%), *aacA4* (5/8, 62.5%) and *bla*_SHV-11_ (4/8, 50.0%). Five transconjugants contained plasmids with the same size as those in their respective donors (TZSKP-1, 9, 15, 146, and 208) (Figure 1). In addition, the transconjugant for TZSKP-82 harbored new plasmid with different size than that in the donor strain. The phylogenetic tree based on the MLST analysis results for the isolates is shown in Figure 2. Seven different STs were identified for those 12 isolates. Three isolates (TZSKP-1, 9, and 82) belonged to ST15, three isolates (TZSKP-13, 15, and 17) belonged to ST11, two isolates (TZSKP-228 and 245) belonged to ST218, and the rest of the isolates had unique STs. The epidemiological links were further determined for the patients from whom the clustered isolates were obtained.

**Table 6:** Transmissibility of drug resistance of ESBL positive MDR, XDR and PDR *K. pneumoniae* isolates by conjugation.

K. *pneumoniae* isolates	Resistance profile of K. *pneumoniae* isolatesa,b	Resistance-associated genes detected in K. *pneumoniae* isolates^b^
MDR isolates		
TZSKP-9	AMP,SAM,CFZ,CRO,CAZ,FEP,ATM,CIP,LVX,SXT,FD	*bla* _CTX-M-14_,*dhfr*
TZSKP-28	AMP,SAM,CFZ,CRO,CAZ,FEP,ATM,GM,TOB,SXT,FD	*bla* _SHV-11_
TZSKP-40	AMP,SAM,CFZ,CRO,CAZ,FEP,ATM,GM,TOB,SXT,FD	*bla*_CTX-M-14_,*bla*_CTX-M-10_,*bla*_SHV-11_,*bla* _TEM-1_,*qnrS,aacC2*
TZSKP-208	AMP,SAM,CFZ,CRO,CAZ,FEP,ATM,SXT,FD	*bla* _CTX-M-9_,*bla*_CTX-M-10_,*bla* _SHV-11_,*qnrS*
XDR isolates		
TZSKP-13	AMP,SAM,CFZ,CRO,CAZ,FEP,ATM,CIP,LVX,TOB,AMK,SXT,FD	*bla* _CTX-M-14_,*dhfr*,*qnrB,aacA4*
TZSKP-15	AMP,SAM,CFZ,CRO,CAZ,FEP,CTT,ETP,IMP,ATM,CIP,LVX,GM,TOB,AMK,SXT,FD	*bla*_CTX-M-1_,*bla* _CTX-M-14_,*bla*_CTX-M-10_,*bla*_SHV-11_,*bla*_TEM_,*dhfr,qnrA,qnrB,aac(6′)-Ib-cr,aacA4,aadA1*
TZSKP-146	AMP,TZP,SAM,CFZ,CRO,CAZ,FEP,ATM,CIP,LVX,GM,TOB,AMK,SXT,FD	*bla*_CTX-M-1_,*bla* _CTX-M-3_,*bla*_CTX-M-9_,*bla*_CTX-M-10_,*bla* _SHV-11_,*bla*_TEM_,*dhfr,qnrD,aac(6′)-Ib-cr,aacA4,aacC2,aadA1*
TZSKP-228	AMP,TZP,SAM,CFZ,CRO,CAZ,FEP,CTT,ETP,IPM,ATM,TOB,AMK,SXT	*bla*_CTX-M-3_,*bla*_CTX-M-10_,*bla*_SHV-11_,*bla*_TEM-1_,*bla*_KPC-2_,*bla*_IMP_,*bla*_VIM_,*bla*_OXA-48_,*dhfr,qnrD,qnrS,aac(6′)-Ib-cr,aacA4,aadB,aphA6*
PDR isolates		
TZSKP-1	AMP,TZP,SAM,CFZ,CRO,CAZ,FEP,CTT,ETP,IMP,ATM,CIP,LVX,GM,TOB,AMK,SXT,FD	bla_CTX-M-3_,bla_CTX-M-10_,bla_SHV-11_,dhfr,qnrB,aac(6′)-Ib-cr,aacA4,aacC2
TZSKP-17	AMP,TZP,SAM,CFZ,CRO,CAZ,FEP,CTT,ATM,CIP,LVX,GM,TOB,AMK,SXT,FD	*bla*_CTX-M-2_,*bla* _CTX-M-14_,*bla*_CTX-M-10_,*bla*_SHV-11_,*bla*_TEM-1_,*bla*_CMY2_,*bla*_DHA1_,*dhfr,qnrS,aac(6′)-Ib-cr,aacA4,aacC2,aadA1*
TZSKP-82	AMP,TZP,SAM,CFZ,CRO,CAZ,FEP,CTT,ETP,IPM,ATM,CIP,LVX,GM,TOB,AMK,SXT,FD	*bla*_CTX-M-3_,*bla* _CTX-M-9_,*bla*_CTX-M-10_,*bla*_OXA-48_,*aac(6′)-Ib-cr,aacA4,aacC2,armA*
TZSKP-245	AMP,TZP,SAM,CFZ,CRO,CAZ,FEP,CTT,ETP,IPM,ATM,CIP,LVX,GM,TOB,AMK,SXT,FD	*bla*_CTX-M-10_,*bla*_TEM-1_,*bla*_KPC-2_,*dhfr,aac(6′)-Ib-cr,aacA4,aacC2*

a Abbreviation of drugs: AMP, Ampicillin; TZP, Piperacillin/Tazobactam; SAM, Ampicillin/Sulbactam; CFZ, Cefazolin; CRO, Ceftriaxone; CAZ, Ceftazidime; FEP, Cefepime; CTT, efotetan; ETP, Ertapenem; IMP, Imipenem; ATM, Aztreonam; CIP, Ciprofloxacin; LVX, Levofloxacin; GM, Gentamycin; TOB, Tobramycin; AMK, Amikacin; SXT, Trimethoprim-Sulfamethoxazole; FD, Furadantin.

b The ones found in both donor strains and transconjugants were underlined to demonstrate the difference.

## Discussion

Worldwide emergence and dissemination of ESBL and carbapenemase genes among *Enterobacteriaceae,* especially in *K. pneumoniae* isolates, poses a considerable threat to public health. The major goal of this study was to evaluate the current situation and genetic background of drug-resistant *K. pneumoniae* isolates from patients in hospital settings. The highest and lowest resistance rates were observed for penicillins (99.6%) and carbapenems (7.2%), respectively. The rates of MDR, XDR and PDR isolates observed in this study are alarmingly high. This could cause difficulty in treating *K. pneumoniae*-associated infections since fewer and fewer effective drugs are available for treating those highly drug-resistant isolates. We also found that the proportion of MDR and other types of cases was significantly higher in patients with ESBL-negative isolates than that for XDR and PDR cases. This could be partially explained by the fact that the ESBL-positive isolates are normally resistant to many drugs, leaving only a few effective drugs available for treatment, which could lead to further resistance to those drugs. Indeed, this study further reveals that resistance to most of the drugs was found to be associated with ESBL positivity. Infections due to those ESBL positive and highly resistant strains are reported to be associated with higher morbidity and mortality rates [19]–[22], thus globally coordinated surveillance of epidemiology of those resistant isolates are warranted.

Another goal of this study was to evaluate the correlation between resistance phenotypes and the genetic determinants clinical *K. penumoniae* isolates, so as to give a “snapshot” of the background of those resistant isolates. A striking feature of this study is the large number of antibiotic resistance-associated genes detected in the examined isolates. We also found that while some of the previously reported resistance-associated genes were indeed detected at relatively higher rates among corresponding phenotypic resistant isolates, some others were detected in very low proportion of the phenotypic resistant isolates, suggesting the existence of unknown drug resistance mechanisms such as reduced permeability of the outer membrane or up-regulated unknown efflux pumps in some clinical isolates [27], [28]. In addition, some of the resistance-associated genes were also detected in a sizable proportion of the phenotypic susceptible isolates, suggesting that individual resistance gene alone is not sufficient to cause resistance phenotype and only when some of them were accumulated can the resistance become detectable in the clinical isolates.

Rapid detection of genetic determinants associated with drug resistance in clinical *K. pneumoniae* isolates is crucial for appropriate antimicrobial therapy and infection control measures. We detected relatively high percentage of previously reported genes associated with resistance to beta-lactams [2], [10], [29]–[32], fluoroquinolones [28], [33]–[37], aminoglycosides [38], [39], and folate pathway inhibitors [15] in *K. pneumoniae* isolates. CTX-M-type beta-lactamase genes (such as *bla*_CTX-M-14_ and *bla*_CTX-M-15_) have been reported to be prevalent worldwide [40]–[42]. For example, a recent study from China reported that among the 21 *K. pneumoniae* isolates from 1270 specimens collected in a prospective multi-center study in eight teaching hospitals in China from June to December in 2007, 3 were detected to have *bla*_CTX-M-14_ (3, 14.3%) [40]. Another study conducted in Scotland showed that 16 of the 219 (7.3%) clinical isolates of *K. pneumoniae* collected in 2006 and 2007 at the Royal Infirmary of Edinburgh, Scotland had *bla*_CTX-M-15_ [41]. In the present study, the highest rate of CTX-M-type beta-lactamase genes was observed for *bla*_CTX-M-10_ (35.9%), followed by *bla*_CTX-M-1_ (16.6%)_,_ *bla*_CTX-M-14_ (16.6%)_,_ and *bla*_CTX-M-15_ (15.2%)_._ The rates of the non-ESBL SHV-type beta-lactamase genes *bla*_SHV-1_ and *bla*_SHV-11_ genes were 24.7% and 21.1%, respectively in this study, which were relatively lower compared to that reported by some previous studies conducted in other regions. For example, according to a study conducted in Korea, the rates of the *bla*_SHV-1_ and *bla*_SHV-11_ genes among *K. pneumoniae* isolates collected from May to July, 2002 were 35% (50/142) and 62% (62/142), respectively [43]. Another study from Brazil reported that 55.8% (29/52) of the *K. pneumoniae* isolates collected in Recife, PE, Brazil during 1998 to 2005 harbored the *bla*_SHV_ genes [44]. Thus, the prevalence of some beta-lactamase genes such as the *bla*_SHV_ genes could be greatly variable geographically and timewise.

The more recently reported carbapenemases genes (such as *bla*_IMP_, *bla*_VIM_, *bla*_NDM_, plasmid-mediated clavulanic acid-inhibited class A beta-lactamases genes such as *bla*_KPC_, and the class D beta-lactamase gene *bla*_OXA-48_) were rarely detected or undetected in this study. Carbapenemases increasingly have been reported in *Enterobacteriaceae* in the past decade. KPC carbapenemases have been reported in the United States and then worldwide [6], [8]. VIM and IMP metallo-beta-lactamases also have been reported in many regions of the world, with a higher prevalence in southern Europe and Asia [1], [5], [45]–[47]. Carbapenemases of the oxacillinase-48 type (OXA-48) have been identified mostly in Mediterranean and European countries and in India [48], [49]. Although the world-alarming New Delhi metallo-beta-lactamase-1 (NDM-1) was not detected in this study, it has been detected worldwide since it was first identified in India and its variants have emerged [13], [50]–[52]. Thus resistance caused by those recently emerging beta-lactamases is still worrisome and needs continuous monitoring. The association of alterations in *gyrA* (gene encoding for GyrA subunit of DNA gyrase) and *parC* (gene encoding for ParC subunit of DNA topoisomerase IV) with fluoroquinolone resistance in *K. pneumoniae* is still not clear. Some studies suggested that in *K. pneumoniae*, DNA gyrase A is a primary target of quinolones and that ParC alterations play a complementary role in the development of higher-level fluoroquinolone resistance [53], [54], while a study reported that hypermutation in *K. pneumoniae* is uncommon and does not contribute to accumulation of *gyrA* mutations or directly to ciprofloxacin resistance [55]. We identified 3 types of *gyrA* mutations including the previously reported C248T (Ser83Phe) and A260C (Asp87Ala) [53] and the unreported T247A (Ser83Ile). No mutations in *parC* were detected in this study. The plasmid-encoded 16 S rRNA methylases *armA* and *rmtB* has emerged as a new mechanism of resistance to aminoglycosides, and the concomitant presence of *armA* or *rmtB* with *bla*_CTX-M_ type beta-lactamase genes, especially the group 1 (CTX-M-3 and CTXM-15) or group 9 (CTX-M-14), among amikacin-resistant ESBL-producing *K. pneumoniae* isolates was reported in Taiwan and Belgium [56], [57]. In this study, both *armA* and *rmtB* were detected (5.8% and 3.6%, respectively). One isolate was found to harbor both *armA* and *rmtB* genes, and consistent with previous reports, the *armA* and *rmtB* genes were coexistent with at least one of the *bla*_CTX-M_ type beta-lactamases tested in this study.

Our study further confirmed the notion that patients infected with carbapenem-resistant *K. pneumoniae* isolates normally have worse treatment outcome. In addition, the conjugation results suggest that certain ESBL genes (such as *bla*_CTX-M-14_), *aac(6′)-Ib-cr* and *aacA4 were* frequently co-transmitted and co-selected in MDR, XDR and PDR isolates and can be naturally transferred to susceptible *E. coli* strains by conjugation. Five transconjugants contained plasmids with the same size as those in their respective donors. Nevertheless, plasmids of the same sizes found in both donor and recipient isolates could not guarantee that the resistance transfer was plasmid-mediated. A subsequent DNA-DNA hybridization experiment with probes made by the respective resistance genes is warranted to show that the plasmids of the same sizes do carry the same resistance genes. On the other hand, we noticed that plasmids were not identified in some of the donor and recipient isolates. This could be explained by the existence of some other non-plasmid-mediated mechanisms involved in the occurrence and transfer of drug resistance in those isolates. For example, the drug resistance-associated genes could be carried on chromosomally located transposons and integrons. Although the isolate TZSKP-28 was detected to be ESBL-positive, only the non-ESBL *bla*_SHV-11_ gene was detected in it. After conjugation experiments, the recipient strain became multidrug resistant and again only the *bla*_SHV-11_ was detected. We also noticed that the beta-lactamase genes found in the transconjugants of the isolates TZSKP-1 and TZSKP-40 (*bla*_SHV-11_ or *bla*_TEM-1_) were not ESBLs, either. This result suggested that some other mechanisms may be involved in causing the ESBL positivity and MDR phenotypes in those isolates. As horizontal transmission event can result in the acquisition of multidrug resistance by wild-type strains, thus this could presumably contribute to the rapid increase in the prevalence of multidrug resistance among clinical bacteria. Another important aspect for infection control is to know whether there is a clonal spread among the highly drug-resistant isolates. Relatively diverse genotypes were identified for those 12 isolates used in conjugation analysis by MLST analysis. Three clusters (which belonged to ST15, ST11, and ST218, respectively) each consisted of 2 or 3 isolates were detected and the epidemiological links were not observed for the patients from whom the clustered isolates were obtained. Further investigation of the transmission patterns of a larger sample of drug-resistant *K. pneumoniae* isolates by MLST analysis is warranted and which is currently underway.

Emerging plasmid-encoded ESBLs and carbapenemases are increasingly reported worldwide [58]. Carbapenemase production encoded by genes located on mobile genetic elements is typically accompanied by genes encoding resistance to other drug classes, and are frequently located on the same mobile DNA elements such as integrons, which act as bacterial recombination systems that mediate the capture and expression of gene cassettes and are considered as the primary mechanism for antibiotic resistance gene acquisition among bacteria and are frequently associated with transposons and conjugative plasmids (http://integrall.bio.ua.pt 2009) [59], [60]. In this study, class 1 integrons were detected in a relatively high percentage of the isolates. Further sequencing analysis of class 1 integrons and gene cassette arrays is currently undergoing in the lab.

In summary, our results indicate that there is a high prevalence and possible transmission of MDR, XDR and PDR *K. pneumoniae* isolates among hospitalized patients. In addition, our data give a “snapshot” of the complex genetic background responsible for drug resistance in those highly drug-resistant *K. pneumoniae* isolates. Thus our study demonstrate that a high degree of awareness and monitoring of those drug resistance determinants are urgently needed in order to better control the emergence and transmission of drug-resistant *K. pneumoniae* isolates in hospital settings.

## Supporting Information

Table S1 **Characteristics of carbapenem-resistant** ***K. pneumoniae*** **isolates.**
(DOC)
