# Supplementary material for: Analysis of Drug Resistance Determinants in Klebsiella pneumoniae Isolates from a Tertiary-Care Hospital in Beijing, China
Source: PLoS One. 2012 Jul 31;7(7):e42280. doi: 10.1371/journal.pone.0042280 (PMC3409176; doi:10.1371/journal.pone.0042280)
Supplement: Table S1 — Characteristics of carbapenem-resistant K. pneumoniae isolates. (DOC) [file pone.0042280.s001.doc]

**Table S1. Characteristics of** **carbapenem-resistant *K. pneumoniae* isolates.**

| **Isolates** | **MIC (μg/mL)** | | **Carbapenemase genes** | **ESBL** | **Resistant phenotype for other antibiotics ^a^** | **Other resistance-associated genes** | **Resistance type** | **Age** | **Gender** | **Diagnosis** | **Specimens** | **Treatment outcome** |
| --- | --- | --- | --- | --- | --- | --- | --- | --- | --- | --- | --- | --- |
|  | ETP | IMP |  |  |  |  |  |  |  |  |  |  |
| TZSKP-1 | ≥8 | ≤1 | None | + | AMP,TZP,SAM,CFZ,CRO,CAZ,FEP,CTT,ATM,CIP,LVX,GM,TOB,AMK,SXT,FD | *bla*_CTX-M-3_,*bla*_CTX-M-10_,*bla*_SHV-11_,*dhfr*,*qnrB*,*aac(6’)-Ib-cr*,*aacA4*,*aacC2* | PDR | 75 | Male | Septic shock, pneumonia | Sputum | Treatment success |
| TZSKP-7 | ≥8 | ≥16 | None | - | AMP,TZP,CFZ,CRO,CAZ,FEP,CTT,ATM,GM,TOB,AMK,SXT,FD | *bla*_CTX-M-14_,*bla*_SHV-11_,*bla*_TEM-1_,*qnrS*,*aacC2* | XDR | 73 | Male | Respiratory failure, cerebral infarction | Sputum | Died |
| TZSKP-17 | ≥8 | ≤1 | None | + | AMP,TZP,SAM,CFZ,CRO,CAZ,FEP,CTT,ATM,CIP,LVX,GM,TOB,AMK,SXT,FD | *bla*_CTX-M-2_,*bla*_CTX-M-14_,*bla*_CTX-M-10_,*bla*_SHV-11_,*bla*_TEM-1_,*bla*_CMY2_,*bla*_DHA1_,*dhfr*,*qnrS*,*aac(6’)-Ib-cr*,*aacA4*,*aacC2*,*aadA1* | PDR | 75 | Male | Urinary tract infection | Sputum | Treatment success |
| TZSKP-25 | ≥8 | ≤1 | None | - | AMP,TZP,SAM,CFZ,CRO,CAZ,FEPCTT,ATM,CIP,LVX,GM,TOB,AMK,SXT,FD | *bla*_CTX-M-1_,*bla*_CTX-M-14_,*bla*_CTX-M-10_,*bla*_SHV-11_, *bla*_CMY2_,*aadA1* | XDR | 75 | Male | Pulmonary infection | Urine | Treatment success |
| TZSKP-82 | ≥8 | ≥16 | *bla*_OXA-48_ | + | AMP,TZP,SAM,CFZ,CRO,CAZ,FEP,CTT,ATM,CIP,LVX,GM,TOB,AMK,SXT,FD | *bla*_CTX-M-3_,*bla*_CTX-M-9_,*bla*_CTX-M-10_,*bla*_OXA-48_,*aac(6’)-Ib-cr*,*aacA4*,*aacC2* | PDR | 81 | Male | Cerebral infarction | Sputum | Died |
| TZSKP-110 | 4 | ≤1 | None | + | AMP,SAM,CFZ,CRO,CAZ,FEP,CTT,ATM,CIP,LVX,TOB,AMK,SXT,FD | *bla*_CTX-M-8_,*bla*_CTX-M-14_,*bla*_CTX-M-10_,*bla*_TEM-1_,*dhfr*,*qnrD*,*qnrS*,*aadA1*,*aacC2* | MDR | 86 | Male | Pneumonia, severe sepsis | Sputum | Died |
| TZSKP-135 | 4 | ≤1 | None | + | AMP,SAM,CFZ,CRO,CAZ,FEP,CTT,ATM,CIP,LVX,TOB,AMK,SXT,FD | *bla*_CTX-M-14_,*bla*_CTX-M-10_,*bla*_SHV-11_,*bla*_TEM-1_,*dhfr*,*qnrD*,*qnrS*,*aac(6’)-Ib-cr*,*aacC2* | XDR | 82 | Male | [Hemorrhagic shock](http://www.google.com.hk/url?sa=t&rct=j&q=失血性休克+英语&source=web&cd=1&ved=0CCUQFjAA&url=http%3A%2F%2Fwww.dictall.com%2Findu%2F069%2F0686036100C.htm&ei=KV1ZT-WnL6aimQWsw8CmDw&usg=AFQjCNG3FGuKBvX2j5VAi9fgWp-s5fS5JQ&cad=rjt) | Pleural effusion | Died |
| TZSKP-161 | ≥8 | ≥16 | *bla*_OXA-48_ | - | AMP,TZP,SAM,CFZ,CRO,CAZ,FEP,CTT,ATM,CIP,LVX,GM,TOB,AMK,SXT,FD | *bla*_CTX-M-1_,*bla*_CTX-M-3_,*bla*_CTX-M-10_,*bla*_SHV-11_,*aac(6’)-Ib-cr*,*aacC1*,*aacC2*,*aadB* | XDR | 58 | Male | Chronic bronchitis | Sputum | Treatment success |
| TZSKP-174 | ≥8 | ≥16 | *bla*_OXA-48_ | - | AMP,TZP,SAM,CRO,CAZ,FEP,CTT,ATM,CIP,LVX,GM,TOB,AMK,SXT,FD | *bla*_CTX-M-1_,*bla*_CTX-M-3_,*bla*_CTX-M-10_,*bla*_SHV-11_,*ac(6’)-Ib-cr*,*aacA4*,*aacC2* | XDR | 57 | Male | Chronic obstructive pulmonary disease | Sputum | Treatment success |
| TZSKP-189 | 4 | ≤1 | None | + | AMP,SAM,CFZ,FD | *bla*_CTX-M-10_,*bla*_CTX-M-25_,*bla*_SHV-11_,*dhfr*,*qnrD* | MDR | 78 | Female | ND ^b^ | Sputum | ND ^b^ |
| TZSKP-197 | ≥8 | 8 | *bla_KPC-2_,bla*_OXA-48_ | - | AMP,TZP,SAM,CFZ,CRO,CAZ,FEP,CTT,ATM,CIP,LVX,GM,TOB,SXT,FD | *bla*_CTX-M-1_,*bla*_CTX-M-3_,*bla*_CTX-M-10_,*bla*_SHV-11_,*bla*_TEM-1_,*dhfr*,*aac(6’)-Ib-cr*,*armA* | XDR | 93 | Male | Chronic bronchitis | Sputum | Died |
| TZSKP- 204 | ≥8 | 4 | *bla*_OXA-48_ | + | AMP,TZP,SAM,CFZ,CRO,CAZ,FEP,CTT,ATM,CIP,LVX,TOB,AMK,FD | *bla*_CTX-M-1_,*bla*_CTX-M-9_,*bla*_CTX-M-10_,*bla*_TEM-1_,*qnrB*,*aac(6’)-Ib-cr*,*aacA4*,*aadA1* | XDR | 73 | Male | Pneumonia | Sputum | Treatment success |
| TZSKP-225 | 2 | 2 | None | - | AMP,TZP,SAM,CFZ,CRO,CAZ,FEP,CTT,ATM,CIP,LVX,TOB,AMK,SXT,FD | *bla*_CTX-M-_*_1_*,*bla*_CTX-M-3_,*bla*_CTX-M-9_,*bla*_CTX-M-10_,*bla*_TEM-1_,*dhfr*,*qnrD*,*aac(6’)-Ib-cr*,*aacA4*,*aacC2*,*aadA1* | XDR | 83 | Male | Chronic obstructive pulmonary disease | Urine | Treatment success |
| TZSKP-228 | ≥8 | 4 | *bla*_KPC-2_*,bla*_IMP_,*bla*_VIM_ | + | AMP,TZP,SAM,CFZ,CRO,CAZ,FEP,CTT,ATM,TOB,AMK,SXT | *bla*_CTX-M-3_,*bla*_CTX-M-10_,*bla*_SHV-11_,*bla*_TEM-1_,*bla*_KPC-2_,*bla*_IMP_,*bla*_VIM_,*bla*_OXA-48_,*dhfr*,*qnrD*,*qnrS*,*aac(6’)-Ib-cr*,*aacA4*,*aadB*,*aphA6* | XDR | 75 | Male | ND ^b^ | Sputum | ND ^b^ |
| TZSKP-240 | ≥8 | 4 | None | - | AMP,TZP,SAM,CFZ,CRO,CAZ,FEP,CTT,ATM,CIP,LVX,TOB,AMK,SXT,FD | *bla*_CTX-M-1_,*bla*_CTX-M-3_,*bla*_CTX-M-8_,*bla*_CTX-M-10_,*bla*_SHV-11_,*bla*_TEM-1_,*dhfr*,*aac(6’)-Ib-cr*,*aacA4*,*aacC2*,*aadA1* | XDR | 56 | Female | Pneumonia | Sputum | Treatment success |
| TZSKP-245 | ≥8 | ≥16 | *bla*_KPC-2_ | + | AMP,TZP,SAM,CFZ,CRO,CAZ,FEP,CTT,ATM,CIP,LVX,GM,TOB,AMK,SXT,FD | *bla*_CTX-M-10_,*bla*_TEM-1_,*bla*_KPC-2_,*dhfr*,*aac(6’)-Ib-cr*,*aacA4*, *aacC2* | PDR | 56 | Male | Acute pancreatitis, shock | Blood | Died |

^a^ Abbreviation of drugs: AMP, Ampicillin; TZP, Piperacillin/Tazobactam; SAM, Ampicillin/Sulbactam; CFZ, Cefazolin; CRO, Ceftriaxone; CAZ, Ceftazidime; FEP, Cefepime; CTT, Cefotetan; ETP, Ertapenem; IMP, Imipenem; ATM, Aztreonam; CIP, Ciprofloxacin; LVX, Levofloxacin; GM, Gentamycin; TOB, Tobramycin; AMK, Amikacin; SXT, Trimethoprim-Sulfamethoxazole; FD, Furadantin.

^b^ No data available.
